# Effect of digital tools in outpatient cardiac rehabilitation including home training—results of the EPICURE study

**DOI:** 10.3389/fdgth.2023.1150444

**Published:** 2023-07-14

**Authors:** Dieter Hayn, Mahdi Sareban, Stefan Höfer, Fabian Wiesmüller, Karl Mayr, Norbert Mürzl, Michael Porodko, Christoph Puelacher, Lisa-Marie Moser, Marco Philippi, Heimo Traninger, Josef Niebauer

**Affiliations:** ^1^ Center for Health & Bioresources, AIT Austrian Institute of Technology GmbH, Graz, Austria; ^2^ Ludwig Boltzmann Institute for Digital Health and Prevention, Salzburg, Austria; ^3^ University Institute of Sports Medicine, Prevention and Rehabilitation, Paracelsus Medical University, Salzburg, Austria; ^4^ Department of Psychiatry II, Medizinische Universität Innsbruck, Innsbruck, Austria; ^5^ CARDIOMED Kardiologisches Rehabilitationszentrum GmbH, Linz, Austria; ^6^ Institut für Präventiv- und Rehabilitationsmedizin, Cardio Vital Wels, Wels, Austria; ^7^ Reha Innsbruck, REHAmed-tirol GmbH, Innsbruck, Austria; ^8^ MedReha GmbH, Feldkirch, Austria; ^9^ ZARG Zentrum für ambulante Rehabilitation GmbH, Graz, Austria

**Keywords:** mHealth, telehealth, cardiac rehabilitation, wearable, adherence

## Abstract

**Introduction:**

Cardiovascular diseases are the leading cause of death worldwide and are partly caused by modifiable risk factors. Cardiac rehabilitation addresses several of these modifiable risk factors, such as physical inactivity and reduced exercise capacity. However, despite its proven short-term merits, long-term adherence to healthy lifestyle changes is disappointing. With regards to exercise training, it has been shown that rehabilitation supplemented by a) home-based exercise training and b) supportive digital tools can improve adherence.

**Methods:**

In our multi-center study (ClincalTrials.gov Identifier: NCT04458727), we analyzed the effect of supportive digital tools like digital diaries and/or wearables such as smart watches, activity trackers, etc. on exercise capacity during cardiac rehabilitation. Patients after completion of phase III out-patient cardiac rehabilitation, which included a 3 to 6-months lasting home-training phase, were recruited in five cardiac rehabilitation centers in Austria. Retrospective rehabilitation data were analyzed, and additional data were generated via patient questionnaires.

**Results:**

107 patients who did not use supportive tools and 50 patients using supportive tools were recruited. Already prior to phase III rehabilitation, patients with supportive tools showed higher exercise capacity (*P*_max_ = 186 ± 53 W) as compared to patients without supportive tools (142 ± 41 W, *p* < 0.001). Both groups improved their *P*_max_, significantly during phase III rehabilitation, and despite higher baseline *P*_max_ of patients with supportive tools their *P*_max_ improved significantly more (*ΔP*_max_ = 19 ± 18 W) than patients without supportive tools (*ΔP*_max_ = 9 ± 17 W, *p* < 0.005). However, after adjusting for baseline differences, the difference in *ΔP*_max_ did no longer reach statistical significance.

**Discussion:**

Therefore, our data did not support the hypothesis that the additional use of digital tools like digital diaries and/or wearables during home training leads to further improvement in P_max_ during and after phase III cardiac rehabilitation. Further studies with larger sample size, follow-up examinations and a randomized, controlled design are required to assess merits of digital interventions during cardiac rehabilitation.

## Introduction

Cardiovascular diseases (CVD) are the leading cause of death worldwide (1) with substantial micro- as well as macro-economic burden (1–3). Several modifiable risk factors contribute to the pathogenesis of CVD which can be addressed during cardiac rehabilitation (CR), i.e., a comprehensive multi-phased secondary prevention framework which has proven to reduce mortality in CVD patients (4) as well as lower its economic burden (5). Increasing physical activity (PA) and exercise capacity are one of the main goals during CR because they have favorable effects on multiple cardiovascular risk factors *(*6) and have shown to be strongly associated with lower mortality in CVD patients (7, 8). As a result, international secondary prevention guidelines advocate PA recommendations, i.e.,150–300 min of moderate intensity or 75–150 min of vigorous exercise each week (9), with higher exercise intensity and duration being associated with greater benefit (10). Notably, the greatest benefits to health and quality of life are likely to be achieved by increases in PA in otherwise sedentary subjects (7, 11), commonly defined as those with <14 METs h/week energy expenditure.

In order to achieve sustainable behavior change, enrollment in CR phase II should take place as soon as possible once a patient meets one of the well-established indications (12). CR phase II can be carried out as in- (IN-II) or outpatient (OUT-II) CR, depending on the severity of the diseases, patients’ preferences, and the availability of an outpatient CR facility in the vicinity. Following IN-II as well as OUT-II, an outpatient phase III (OUT-III) enrollment is offered with weekly visits at the outpatient CR facility to maintain short-term lifestyle changes.

Prior to and after OUT-III, detailed examinations are performed in the CR facilities, and include, among others, questionnaires, anthropometric assessment, blood tests, resting and exercise ECG.

Despite its proven merits, sustainable behavior change, i.e., long-term preservation of recommended PA volume and exercise capacity following completion of CR phase II, is disappointing (13, 14). This gap is addressed in recent recommendations (9), suggesting considering the use of consumer-based wearable activity trackers to increase PA participation and long-term adherence to healthy behaviors. Especially during OUT-III, supportive tools like digital training diaries and/or commercially available wearable devices might be of help for patients and clinicians to support patients during their home-training phase.

During CR, digital tools can be applied in numerous ways. Patients can use training diaries to document training sessions, receive reminders, etc., monitor vital parameters like heart rate during training sessions, monitor physical activity with activity trackers, or engage in comprehensive tele-rehabilitation programs. In the past decade, various studies and review articles concerning the effect of tele-rehabilitation and supportive tools during tele-rehabilitation on cardiac patients have been published. Recently, five review articles (15–19) analyzed 81 different original articles (four original articles were analyzed by four of the five reviews, four articles by three reviews, and 16 articles by two of the reviews). Tele-rehabilitation proofed to be feasible and acceptable, and it showed similar or superior effectiveness in terms of exercise capacity, physical activity, and/or adherence to CR. While there seem to be various benefits of tele-rehabilitation as compared to regular CR, tele-rehabilitation was found to bear only a very low risk for adverse events (20). Finally, tele-rehabilitation was found to be as cost-effective as center-based CR (21).

The studies described above applied digital support to patients in various settings and phases of CR. Only one study (22) assessed in these reviews included Austrian data. However, this study described a field experiment with 29 male patients in a specific test setting. Although we assume that the use of digital tools has a positive impact on cardiac rehabilitation in Austria, no evidence is available so far.

In our multi-center study, we analyzed the effect of digital tools on exercise capacity during OUT-III cardiac rehabilitation including a home training phase in Austria.

## Materials and methods

### Study design

We assessed the effect of multi-modal supportive tools on exercise capacity during OUT-III CR including home training in the multi-centric EPICURE study that was performed in five outpatient CR centers in Austria. In general, Austrian rehabilitation centers adhere to the latest national and international recommendations for center- and home-based exercise training (12). These recommendations emphasize the importance of personalized exercise training prescription after performing initial medical evaluations including exercise testing and risk assessment. The study protocol was approved by the ethics committee of Upper Austria (vote nr. 1165/2019) and registered at ClincalTrials.gov (Identifier: NCT04458727).

The primary objective was to investigate the effect of the patients’ preference-based choice of supportive tools (phone-based assessments, digital training diaries with/without adherence monitoring and with/without wearables) on the change of maximum power during ergometry pre and post CR phase OUT-III including home training. Secondary objectives included subgroup analyses concerning the effect of each of the supportive tools separately.

Since patients were recruited retrospectively, i.e., after OUT-III, blinding or randomization was not feasible.

### Recruitment

Consecutive patients performing their examination post OUT-III including home training in one of the CR centers were screened for in- and exclusion criteria. In case of eligibility, willingness to participate in the study and after written informed consent was obtained, patients were included in the study and available data from the beginning of CR until inclusion into the study were analyzed.

**Figure 1:**
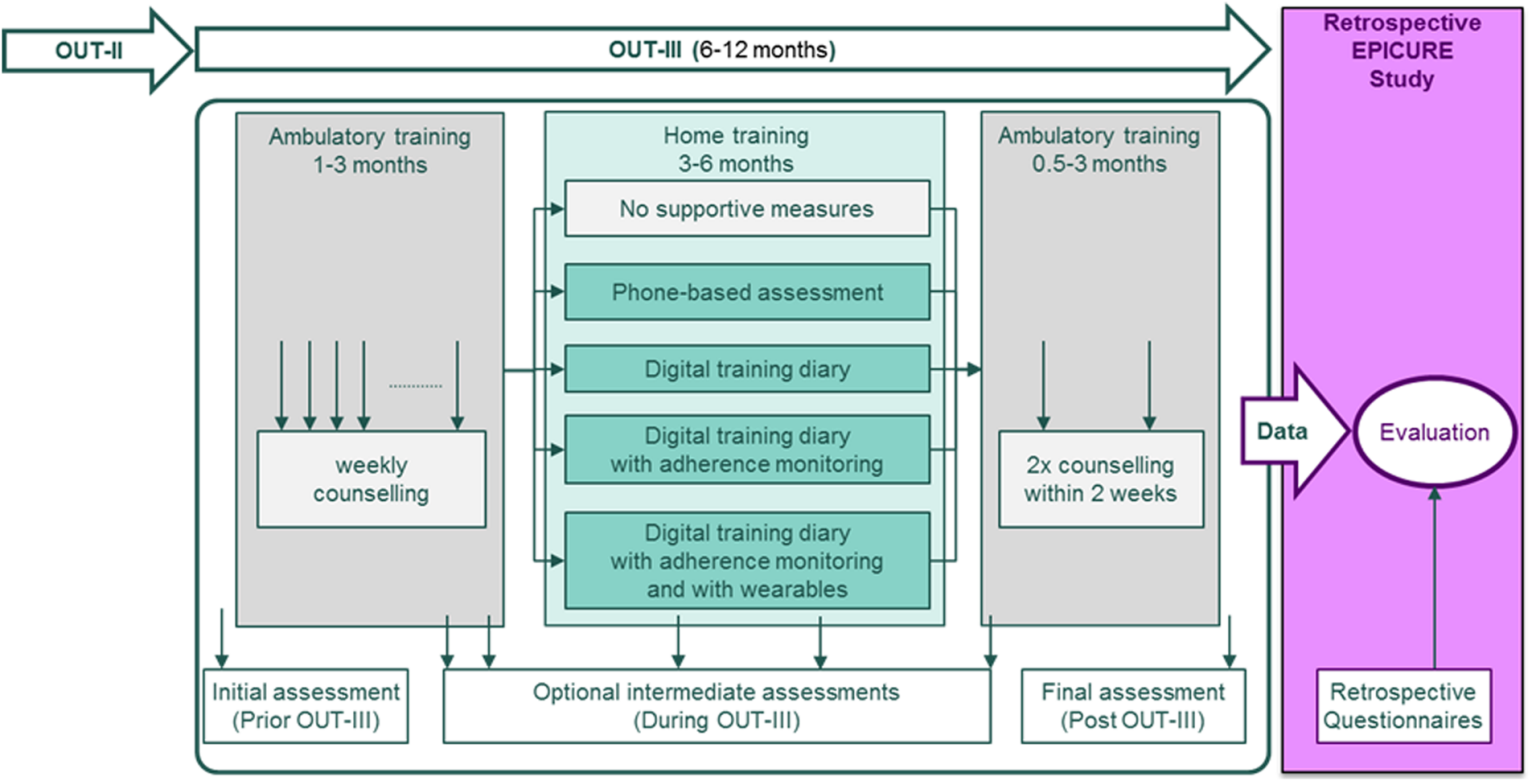
Illustration of the rehabilitation OUT-III phase and the timeline of the EPICURE study.

The CR timeline, the time period analyzed for the primary hypothesis, and the timepoint for inclusion in the EPICURE study are illustrated in Figure 1.

Inclusion criteria:

• Age ≥ 18 years.
• Participation in a phase III rehabilitation at one of the study sites.
• Documented cardiovascular disease.
• The patient is able to give consent to participate in the study.
• Written informed consent to participate in the study.

Exclusion criteria:

• none.

Each patient was assigned to one of two groups, based on their respective answers in the questionnaires:

• Patients who used at least one of the following supportive tools during their home training phase
  ○ Regular phone-based assessment.
  ○ Digital training diary with or without adherence monitoring and with or without wearables.
• Patients who used none of the above-mentioned supportive tools.

### Data acquisition

Two types of data were recorded:

• Retrospective, pre-existing data as collected during CR, including demographic and clinical data, and quality of life according to MacNew-27 (23).
• Questionnaires concerning CR that were filled out by the patient after signing informed consent.

### Statistical analysis

We analyzed the maximum workload *P*_max_ [W] as achieved by patients during the exercise stress test at their regular assessments in the study centers. The difference *ΔP*_max_ [W] between *P*_max_ at the end of OUT-III minus *P*_max_ at the beginning of OUT-III was determined. If no data from the assessment prior to OUT-III was available, data from the assessment post phase II was taken instead. Normal distribution of the data was tested by using the Shapiro-Wilk test. A student t-test was applied to test for global differences between pre- to post CR (dependent *t*-test) and for differences between the groups (independent *t*-test). A value of alpha <0.05 was considered significant. Additionally, we applied ANCOVA statistics to correct on those variables that significantly differed in the two groups.

### Primary hypothesis and sub-group analyses

The primary hypothesis was that *ΔP*_max_ [W] of patients who used any of the above-mentioned supportive tools was significantly higher (*p* < 0.05) as compared to *ΔP*_max_ [W] of patients without supportive tools.

In addition, we analyzed differences between sub-groups of patients with supportive tools to better understand the influence of regular phone-based assessments, digital training diaries alone and diaries in combination with adherence monitoring and wearables, i.e., heart rate monitoring during training and activity trackers. Therefore, we defined two sub-groups (A and B) per supportive tool and the group C without supportive tools:

A. Patients with digital training diary and with the respective supportive tool.
B. Patients with digital training diary and without the respective supportive tool.
C. Patients without supportive tools.

We compared patients with digital training diary and the respective supportive tool (group A) to (a) patients without a supportive tool (group C) and (b) all patients not included in group A (i.e., groups B + C). Therefore, ANCOVA was applied, taking into account all significantly different baseline parameters for the respective group assignment.

### Power calculation

The effect size of 0.46 was derived from mean power and standard deviation at the end of a standard CR program (24). Assuming a minimal clinically important difference of 25 W (25), an *α*-error probability of 0.05, and a power (1-*β* error probability) of 0.8, the total sample size for the 2 groups analyzed by an unpaired *t*-test was determined as 150 participants, i.e., 75 per group.

## Results

### Patient characteristics

Details concerning patient characteristics of all recruited patients as recorded prior to OUT-III are presented in Table 1. Approximately two third of patients reported to have used supportive tools, as specified above, during their home training. This ratio was similar for men and women. Patients using supportive tools were significantly younger, fitter (in terms of *P*_max_), had a lower BMI and body weight, and reported a higher quality of life in all 4 aspects of the MacNew questionnaire prior to OUT-III.

**Table 1:** Patient characteristics.

	Without supportive tools	With supportive tools	*p*-value
Number of patients (female)	107 (30)	50 (11)	
Age	**62** ± **9 y**	**55** ± **13 y**	**<**.**001**
Maximum power during ergometry (*P*_max_)	**142 **± **41 W**	**186 **± **53 W**	**<**.**001**
Body mass index (BMI)	**27.9 **± **4.7 kg/m^2^**	**26.4 **± **4.4 kg/m^2^**	**0**.**038**
Body weight	86 ± 16 kg	82 ± 13 kg	0.130
Non-smoker / ex-smoker / smoker	28 / 46 / 14	18 / 23 / 1	0.058
Blood pressure			
Systolic	118 ± 11 mmHg	120 ± 17 mmHg	0.771
Diastolic	77 ± 8 mmHg	75 ± 8 mmHg	0.454
Lab			
Glucose	105 ± 19 mg/dl	99 ± 30 mg/dl	0.230
LDL cholesterol	85 ± 32 mg/dl	77 ± 35 mg/dl	0.227
HDL cholesterol	**48** ± **12 mg/dl**	**52 **± **12 mg/dl**	**0**.**042**
Triglycerides	115 ± 53 mg/dl	99 ± 59 mg/dl	0.159
MacNew			
Global	**5.68 ** **±** ** 0.88**	**6.11 ** **±** ** 0.76**	**0**.**017**
Physical	**5.64 ** **±** ** 0.93**	**6.15 ** **±** ** 0.81**	**0**.**008**
Emotional	**5.57 ** **±** ** 0.91**	**6.01 ** **±** ** 0.80**	**0**.**019**
Social	**5.84 ** **±** ** 0.91**	**6.23 ** **±** ** 0.87**	**0**.**043**

Significantly different parameters (*p* < 0.05) are highlighted in bold.

### Exercise capacity pre and post OUT-III

 Table 2 summarizes all exercise capacity data achieved pre and post OUT-III for patients with and without supportive tools, including differences and *p*-values.

**Table 2:** Maximum power during ergometry (P_max_) pre and post OUT-III.

	All	Pre	Post	*p*-Value
All	162.08 ± 51 W	156.20 ± 49 W	167.97 ± 55 W	<0.001
Without supportive tools	146.93 ± 43 W	142.42 ± 41 W	151.45 ± 47 W	<0.001
With supportive tools	195.01 ± 55 W	185.63 ± 53 W	204.40 ± 58 W	<0.001
*p*-value	<0.001	<0.001	<0.001	–

**Figure 2:**
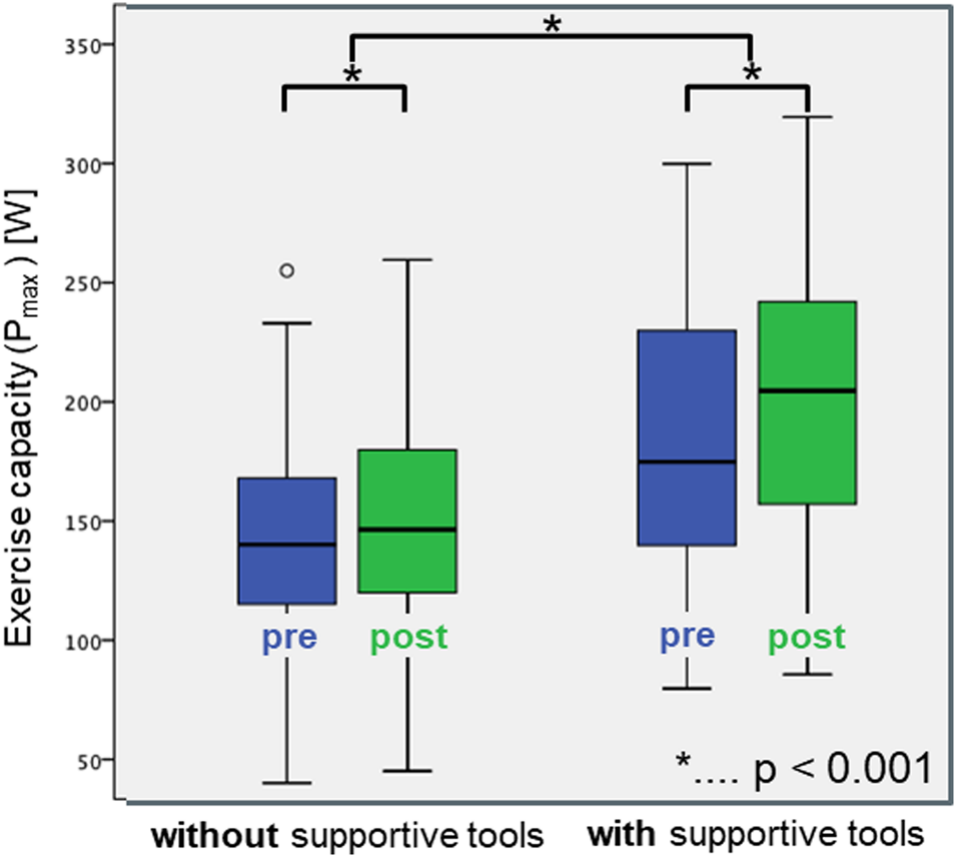
Boxplots of the maximum power during ergometry pre (blue) and post (green) OUT-III for patients with and without supportive measures.

Figure 2 illustrates the maximum power achieved during ergometry pre and post OUT-III for both study groups. *P*_max_ was higher post OUT-III as compared to pre OUT-III in patients with supportive tools, without supportive tools and for the whole study population. *P*_max_ was higher in patients using supportive tools as compared to patients without supportive tools, prior to and post OUT-III and when combining results pre and post OUT-III.

Although patients with supportive tools already showed a higher exercise capacity prior to OUT-III, *P*_max_ improved significantly more (19 ± 18 W) during OUT-III as compared to patients without supportive tools (9 ± 17 W, *p* < 0.005).

**Figure 3:**
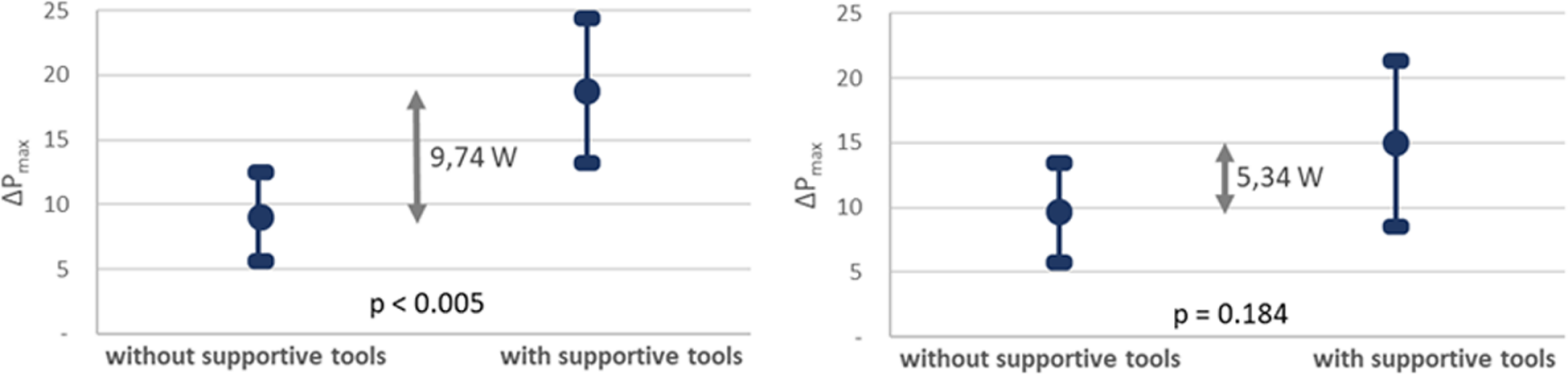
Mean value and confidence interval of the change of maximum power during ergometry pre and post OUT-III (*ΔP*_max_) for patients with and without supportive measures without (**left**) and with (**right**) ANCOVA correction on co-variates.

Five variables differed significantly between the two groups pre OUT-III: age, *P*_max_, HDL cholesterol, quality of life according to MacNew, and BMI. When adjusting for the difference of the variables between the two groups using analysis of covariance according to ANCOVA, the difference in *ΔP*_max_ between the groups was reduced to 5* *± 4 W and did no more reach statistical significance (*p* = 0.184). The change of exercise capacity for both groups with and without ANCOVA correction is illustrated in Figure 3 (results are plotted as mean +/- confidence intervals, since boxplots are not applicable after ANCOVA correction).

### Sub-group analyses

Details concerning sub-group analyses applied to the group with supportive tools are summarized in Table 3. None (0%) of the patients was monitored by regular phone-based assessments, while all 50 patients did use a digital training diary. 17 out of those 50 patients (34%) reported to have had adherence monitoring during home training. 46 out of 50 patients (92%) with a digital training diary were also using wearables. All these 46 patients monitored their heart rate during training. 41 out of 50 (82%) patients additionally used an activity tracker to monitor physical activity.

**Table 3:** Subgroup analysis including number of patients per subgroup A, B and C, *p*-value between subgroups for the change *ΔP*_max_ in the maximum power during ergometry (*P*_max_) at the end of OUT-III minus *P*_max_ at the beginning of OUT-III.

	Data per sub-group			*p*-value	
Tool	Digital diary with respective tool (A)	Digital diary without respective tool (B)	No supportive tool (C)	A vs. C	A vs. B + C
Regular phone-based assessments	n.a.	18.77 ± 2.77 W	9.03 ± 1.73 W	n.a.	n.a.
	(0)	(50)	(107)		
Adherence monitoring	12.67 ± 3.73 W	22.03 ± 3.66 W	9.03 ± 1.73 W	0.716	0.886
	(17)	(33)	(107)		
Heart rate monitoring	19.90 ± 2.90 W	3.67 ± 1.86 W	9.03 ± 1.73 W	0.060	**<0**.**05**
	(46)	(4)	(107)		
Activity tracker	16.03 ± 2.49 W	30.75 ± 9.57 W	9.03 ± 1.73 W	**<0**.**05**	0.241
	(41)	(9)	(107)		

Data concerning sub-groups A, B and C are represented as mean *ΔP*_max _± standard deviation (number of patients). *p*-values were calculated with ANCOVA analysis.

*p*-values 0.05 are highlighted in bold.

We compared patients with digital training diaries and the respective supportive tool (group A) to (a) patients without a supportive tool (group C) and (b) all patients not included in group A (i.e., groups B + C). Adherence monitoring did not have a significant influence on *ΔP*_max_ in neither of these analyses.

A significant difference between patients using a digital training diary in combination with heart rate monitoring during training compared to all other patients (A vs. B + C, *p* < 0.05) was found. We also observed a trend in *ΔP*_max_ between patients with diary and heart rate monitoring as compared to those without digital training diaries, however, the difference did not reach statistical significance (A vs. C, *p* = 0.060).

*ΔP*_max_ significantly differed between patients with digital training diaries and activity tracker as compared to patients without digital training diaries (A vs. C, *p* < 0.05). No difference in *ΔP*_max_ between patients with digital training diaries and activity tracker as compared to all other patients was found (A vs. B + C, *p* 0.241).

## Discussion

Patients in both groups significantly improved their exercise capacity during OUT-III including home-based training. Supportive tools were more frequently used by younger patients, with higher exercise capacity, lower HDL-cholesterol, lower BMI, and better quality of life prior to OUT-III. Patients who used supportive measures during the home training phase of OUT-III improved their exercise capacity more than patients who did not use any supportive measures. However, when applying ANCOVA to consider significant baseline differences in the study groups, the difference in *ΔP*_max_ between the two groups did no longer reach statistical significance.

Subgroup analyses revealed that digital training diaries in combination with heart rate monitoring during training or activity trackers led to significantly better improvement of exercise capacity in some subgroups (see Table 3). However, since in some groups, large variations between individual patients were identified, and since the number of patients in some sub-groups was low, further prospective studies in larger cohorts are indicated to analyse these effects.

Our results are in accordance with previous studies (15–19). However, to the best of our knowledge, the effect of using supportive tools during OUT-III that includes a home-based training phase has not been analysed so far. Our results confirm that OUT-III is effective. Additionally, the use of digital tools has the potential to support sustainable behaviour change and to further improve the exercise capacity of cardiac patients during CR, although our results did not reach statistical significance.

We did not check the use of digital tools but trusted in the patients’ respective answers and we did not consider whether adherence monitoring was really applied or the extent to which patients used supportive tools, which may have led to a rather heterogeneous group with digital tools. These facts need to be kept in mind when interpreting our results.

Although questionnaire data was recorded prospectively, most data in our study were analysed in a retrospective setting based on pre-existing routine-care data, including our primary outcome, i.e., maximum exercise capacity. Additionally, patients were not randomised to the two groups, but they declared within the questionnaire whether they used digital tools or not.

Initially, we planned to identify 50% of patients in the group with and 50% without digital tools. However, since no stratification or randomization was applied, we finally ended up with approx. one third with and two third of patients without digital tools, which reduced the power of our analyses. Therefore, since the number of patients using digital tools was rather low, additional studies with larger sample size may be indicated to confirm our results. To find out more about the effect of supportive tools as compared to the differences in baseline parameters on the training effect, a prospective study may be necessary, where patients are randomized to either a group with or without supportive tools, preferably including stratification on the above-mentioned baseline parameters.

## Conclusion

Our results demonstrate an improvement in exercise capacity post OUT-III cardiac rehabilitation, which included a 3 to 6 months home-training phase as compared to pre OUT-III. Data from patients using supportive tools during OUT-III give first indications that an even greater increase in exercise capacity is possible, suggesting that supportive digital tools might help improve and/or maintain physical exercise capacity by supporting sustainable behaviour change.

## Data Availability

The datasets presented in this article are not readily available because based on study protocol, ethics approval and informed consent, we may only give access to the collected data to authorized personnel, as required for analyzing the data. Requests to access the datasets should be directed to DH, dieter.hayn@ait.ac.at.
